# Unraveling the Intricacies of Powdery Mildew: Insights into Colonization, Plant Defense Mechanisms, and Future Strategies

**DOI:** 10.3390/ijms26083513

**Published:** 2025-04-09

**Authors:** Chun-Mei Gan, Ting Tang, Zi-Yu Zhang, Mei Li, Xiao-Qiong Zhao, Shuang-Yu Li, Ya-Wen Yan, Mo-Xian Chen, Xiang Zhou

**Affiliations:** 1 Key Laboratory of Plant Resource Conservation and Germplasm Innovation in Mountainous Region (Ministry of Education), College of Life Sciences/Institute of Agro-Bioengineering, Guizhou University, Guiyang 550025, China; 18235295082@163.com (C.-M.G.); 13118503124@163.com (X.-Q.Z.); 2 State Key Laboratory of Green Pesticide, Key Laboratory of Green Pesticide and Agricultural Bioengineering, Ministry of Education, Center for R&D of Fine Chemicals, Guizhou University, Guiyang 550025, China; organictt@163.com (T.T.); m18334068576@163.com (Z.-Y.Z.); lm632311531@163.com (M.L.); shilishuangyu@163.com (S.-Y.L.); yanyawen@gmail.com (Y.-W.Y.)

**Keywords:** biopesticide, pathogen, post-transcriptional regulation, powdery mildew, symbiotic microorganism

## Abstract

Powdery mildew, a debilitating phytopathogen caused by biotrophic fungi within the order *Erysiphales*, endangers crop yields and global food security. Although traditional approaches have largely emphasized resistant cultivar development and chemical control, novel strategies are necessary to counter the advent of challenges, such as pathogen adaptation and climate change. This review fully discusses three principal areas of pathogen effector functions, e.g., the reactive oxygen species (ROS)-suppressive activity of CSEP087, and host susceptibility factors, like vesicle trafficking regulated by Mildew Locus O (MLO). It also briefly mentions the transcriptional regulation of resistance genes mediated by factors, like WRKY75 and NAC transcription factors, and post-transcriptional regulation via alternative splicing (As). In addition, this discussion discusses the intricate interactions among powdery mildew, host plants, and symbiotic microbiomes thereof, highlighting the mechanism through which powdery mildew infections disrupt the foliar microbiota balance. Lastly, we present a new biocontrol approach that entails synergistic microbial consortia, such as combinations of *Bacillus* and *Trichoderma*, to induce plant immunity while minimizing fungicide dependency. Through the study of combining knowledge of molecular pathogenesis with ecological resilience, this research offers useful insights towards climate-smart crop development and sustainable disease-management strategies in the context of microbiome engineering.

## 1. Introduction

Powdery mildew is a broad-spectrum plant pathogen that falls within the varied *Ascomycetes*, *Heteromycetes*, and *Powdery Mushrooms*. These biotically dependent fungi that are highly specialized in their nutritional requirements have been reported to infect over 10,000 species of plants that include cultivated, as well as wild, crops. The prevalence of this fungal disease significantly minimizes the yields of most plants, leading to significant economic loss [1,2,3,4,5,6]. The worldwide agricultural sector is increasingly being challenged by the growing incidence of powdery mildew, estimated to impose economic losses worth 6.3 billion USD [7,8]. The severity of the economic losses highlights the risk of fungal disease to a wide range of plants. This is exemplified by the application of propiconazole (PBZ) to enhance cucumber resistance to powdery mildew and wheat breeding for varieties with inherent resistance traits [9,10]. The effectiveness of these methods is increasingly undermined by pathogen evolution, such as the emergence of fungicide-resistant alleles, and unforeseen ecological effects, such as disruption of the soil microbiome [11,12]. For example, MLO-knockout wheat lines remain resistant to *Blumeria graminis* for long periods, while in other crops’ equivalent resistance mechanisms, more often than not, they fail due to functional redundancy in susceptibility networks [13]. These hindrances highlight the urgent need for new, system-level strategies in research on plant–pathogen interactions and to develop long-term solutions.

One unifying hypothesis for modern research is that effective fungal colonization rests on a two-part strategy: (1) immune suppression mediated by effectors [14]; and (2) the disruption of host susceptibility pathways [15]. The molecular mechanisms underlying the pathogen–host interaction, however, are not well understood, particularly in the context of the dynamic processes of transcriptional reprogramming and post-transcriptional regulation that occur during infection. The most recent studies pinpoint AS as a critical, yet under-emphasized, element of plant immune regulation. The networks governing stress-induced gene expression are widely documented to impact downstream gene expression [16]. AS, being a post-transcriptional process, generates various protein isoforms with different functions to generate a quick response to biotic stress [17]. For example, *MeSCL33*, a cassava splicing factor, controls ABA biosynthesis through the splicing of *MeABA1* pre-mRNA to retard postharvest physiological deterioration [18]. LUC7, a U1 snRNP protein component, guides stress-induced AS in *Arabidopsis*, and mutants of such are hypersensitive to abiotic stress, such as salt and cold [19]. The interface between transcriptional regulation and AS is central to the modulation of defense mechanisms. It is currently understood that AS significantly enhances plant immunity to environmental stress, particularly via immune receptor gene regulation and components of defense signaling [20]. The identification and characterization of splicing factors and the identification of alternatively spliced transcripts can potentially yield valuable information on the regulation of plant defense against powdery mildew [21,22,23].

The review focuses on the studies of new defense mechanisms, i.e., transcriptional regulation, AS, and epigenetic regulation, and how they play a role in the reinforcement of plant resistance to powdery mildew. The aim is to provide a comprehensive discussion of these mechanisms and their application in the prevention of powdery mildew. To ensure comprehensiveness, a thorough search of the literature was carried out using PubMed and Web of Science between 2010 and 2025, using the keywords “mildew pathogenesis”, “effector proteins”, “alternative splicing”, and “plant immune”. Such a comprehensive analysis is particularly relevant following the recent breakthrough finding of various microbiome-editing tools, which will allow for new biological control methods that move beyond the classical host–pathogen model.

## 2. Traditional Research on Powdery Mildew

Classical studies on powdery mildew have formed the basis of our knowledge of this fungal disease. Powdery mildew is a widespread disease that attacks a large number of plants, both monocots and dicots [24,25]. Much of the earlier work in this area dealt with the identification of resistant cultivars and breeding strategies [26,27]. Stacking resistance genes, pairing MLO with other disease-resistance genes to boost defense, and targeting known susceptibility genes for knockout are a few of the strategies that have been utilized in developing novel plant varieties. In recent times, there is an increasing emphasis on the complex interactions between pathogens and their host plants.

We continue to have poor knowledge of the complex molecular mechanisms that regulate the growth and development of fungal pathogens. One of the primary challenges is that these pathogens exist only in host tissues, and it is difficult to observe their behavior outside of the host context [28]. Therefore, studying the pathogenesis of powdery mildew remains a significant challenge, and many aspects are not yet well understood. In this article, we briefly summarize the known mechanisms of powdery mildew pathogenesis. For a more in-depth analysis, refer to Table 1, which shows related research.

### 2.1. Symptoms and Effect of Powdery Mildew Infection on Plants

#### 2.1.1. Symptoms of Powdery Mildew Infection

Powdery mildew disease manifests itself as whitish-to-gray powdery spots on various plant parts, like the leaves, stems, flowers, and fruits [37]. Aside from these visible spots, the disease may bring about other signs [38]. Leaves attacked by powdery mildew may exhibit curling or distortion, which hinders their proper growth and formation [39]. Plants that are affected by this disease may experience stunted growth resulting in reduced height and general development [40]. The powdery mildew also prevents the synthesis of chlorophyll, causing the yellowing or browning of leaves [41]. In advanced cases, the disease causes early leaf drying and shedding, impairing photosynthesis and nutrient movement. Powdery mildew-diseased fruits typically have a decrease in quality, which has the potential to lower the total yield of the plant [42].

The symptoms collectively cause a weakening of plant health, which has a negative impact on the yield of the crop. Early detection and management of the symptoms minimize both economic loss and cosmetological damage as a result of powdery mildew infection.

#### 2.1.2. Effects of Powdery Mildew Infection on Plants

Powdery mildew is a very dangerous disease for plant health with very harsh implications for the plants themselves, as well as farmers’ financial security. The formation of powdery spots on leaves inhibits light, which is necessary for photosynthesis. This decrease in carbon fixation has negative impacts on the biomass of the plants and can check growth and overall vigor. It can even kill plants in worst-case scenarios [43]. In addition, powdery mildew compromises the immune system of the plant, leaving it vulnerable to other disease and environmental factors. Such heightened vulnerability can lead to further health problems for the plant [12,44]. Additionally, the presence of powdery mildew hinders the intake and assimilation of nutrients, adversely affecting the nutritional well-being of the plant [45]. Such a condition can lead to nutrient deficiencies, thereby compromising the healthiness of the plant.

### 2.2. Pathogenesis of Powdery Mildew at Different Stages

#### 2.2.1. Fungal Spore Germination and Appressorium Formation

The asexual life cycle of powdery mildew is initiated when ascospores come to rest on a plant’s surface [46]. In *Bgh*, for example, the fungus secretes epidermal alkanes or long-chain aldehydes, which may serve either to adhere to the leaf surface or as signals for the morphogenetic development associated with infection by powdery mildew. The disease is spread by infective SPs that bear conidia. Loss-of-function MLO alleles are responsible for conferring powdery mildew resistance; however, the pathogenicity of the fungus is unchanged throughout its evolutionary history [47].

Throughout each stage of infection, the fungus interacts with the host plant, and this interaction leads to the development of disease. It is essential to understand the process of the disease throughout these stages for developing effective control measures. It has concentrated its studies on the penetration of the fungus into the plant, haustorial growth in the plant, and infectious SP dispersal. The fungi first form an appressorium to penetrate into the plant, research has established. They have also identified fungal effectors—molecules that help the fungus in bypassing the plant defense system [48]. Haustoria, the structures formed by fungi within plant cells, are responsible for the uptake of nutrients. The research indicates that the fungus’s small secreted proteins play a role in the formation of these structures by modulating the plant’s immune responses [48]. Pathogenesis at various stages of powdery mildew involves intricate interactions between the host plant and the fungus, which can include the mutual exchange of noncoding small RNAs. Cross-border RNA interference and noncoding RNA fragments can have site-specific action [12]. Experiments on penetration processes, haustoria development, and the production of infectious SPs have shed light on the molecular mechanism of the disease. Further studies in this direction will facilitate effective measures for controlling and managing powdery mildew.

#### 2.2.2. Mechanisms and Factors Influencing Powdery Mildew Pathogenesis

Powdery mildew has a serious impact on numerous cash crops and ornamental crops [49]. Knowledge of the pathogenesis of powdery mildew, including all the important stages, is key to organizing the appropriate management strategies.

The process begins when fungal SPs are deposited onto the plant surface [50]. These SPs develop specialized structures that adhere to the plant. The SPs proceed to form specialized structures that cling to the plant, imposing mechanical stress and secreting enzymes that aid in breaking through the layers of protection of the plant, including the stratum corneum and epidermal cells [51]. After the fungi infect the plant tissues, they develop haustoria, which invade host cells and induce the production of special feeding structures called haustorial mother cells. These cells facilitate nutrient transfer from the host to the parasite, supporting the growth and reproduction of fungi [52]. Powdery mildew contains a fungal ribonuclease-like effector protein, CSEP0064/BEC1054, which interferes with plant immunity and inhibits the degradation of host ribosomal RNA [53]. Powdery mildew fungi in *Arabidopsis* and barley accumulate specific proteins, such as MLO, which play a crucial role in MLO resistance during infection of the host cells. The proteins play a significant role in pathogenicity since they regulate vesicle-associated processes at the periphery of the plant cell [54,55]. RACE1, a strain of Japanese *Bgh*, can overcome the mlo-mediated resistance in host cells where there is downregulated expression of the Mildew resistance Locus O [56]. Multitudinous virulence factors are involved in powdery mildew pathogenesis. As an example, the primary pathogen of powdery mildew in peas, *Erysiphe pisi* (*Ep*), has three of its newly found virulence factors, *EpCSEP001*, *EpCSEP009*, and *EpCSP083*, which play a central role in pathogenesis [57]. Powdery mildew will not typically kill the host plant but can cause extensive damage, especially to leaves, petioles, and stems, with potentially severe economic and agricultural consequences [58]. Additionally, the presence of other microorganisms in the plant microbiome can influence the establishment and growth of powdery mildew [53]. The interactions among different microorganisms in the plant microbiome can alter the effects of powdery mildew infections [59].

Knowledge of the mechanisms and factors implicated in powdery mildew pathogenesis is essential for effective disease control. This work supports plant defense mechanisms with an ultimate improvement in crop quality and increased yields.

### 2.3. Factors Affecting the Colonization of Powdery Mildew Pathogen

Factors affecting the colonization of powdery mildew pathogens play a crucial role in the development and severity of the disease. Understanding these factors is essential for devising effective strategies to control and manage powdery mildew out-breaks.

#### 2.3.1. Environmental Factors

Powdery mildew diseases favor specific temperature levels [60]. Extremely high or low temperatures tend to reduce the growth of such fungi [61]. High humidity and moisture at the surface also enhance SP germination and growth of the fungus, thereby contributing to powdery mildew formation [62]. In order to avoid the risk, there should be proper air flow and regulation of the moisture content. Vegetables that are in shaded or heavily canopied locations may be more susceptible to powdery mildew infections, as impaired air circulation and light may favor the disease [63]. Moreover, the effect of high CO_2_ levels on the interaction between cucurbit plants—such as squash, cucumbers, and melons—and powdery mildew fungi is a new area of investigation in the broader context of climate change and agricultural management [64].

#### 2.3.2. Host Plant Factors

Proper nourishment is important for a plant’s capacity to resist powdery mildew [65]. Healthy plants that are well-nourished can be expected to possess more robust disease defense mechanisms, as a plant’s genetic predisposition also governs the plant’s susceptibility to powdery mildew. There are some plants that have mechanisms of resistance that enable them to recognize the pathogen and trigger a strong defense response, perhaps mediated by the expression of resistance genes [66]. Plant breeders normally select resistant genotypes to develop cultivars that are resistant to powdery mildew [67]. There are, however, certain powdery mildew races or strains that evade plant resistance successfully, so genetic diversity is important in breeding programs. Moreover, plant hormonal signaling networks are the core of their pathogen defense.

SA and jasmonic acid (JA) are the main hormones involved in these defense mechanisms [68,69]. The interaction and dynamics between these hormones may influence a plant’s susceptibility to pathogens [70]. These hormonal interactions could influence the development of powdery mildew. Each hormone is associated with particular defense responses: SA is primarily associated with defense against biotrophic pathogens, while JA and ethylene (ET)are more commonly associated with defense against necrotrophic pathogens. The coordinated action of these phytohormones indicates the dynamic and plastic nature of plant defense responses, enabling plants to react effectively to multiple environmental stresses.

#### 2.3.3. Physiological Factors

Besides the extrinsic factors, plant pathogens can have an immense influence on the infected plants. Effector proteins, for example, play an essential part in such activities [71]. Young plant tissues undergoing active development, such as young leaves and buds, are particularly susceptible to powdery mildew infection. Their persistent growth explains why such increased susceptibility could occur since such processes may slow down the full induction of their defense system. Experiments have shown that actin rearrangement enables the host nucleus to be transported toward the site of fungal penetration, hence facilitating successful colonization in compatible *Pisum sativum*–powdery mildew interactions [72]. The infection-induced *Erysiphe* necator NAFU1 effector CSEP087 contains a signal peptide, implying that it could be secreted into plant cells. Its action in grapevine leaves enhances the proliferation of powdery mildew by inhibiting the production of ROS [73]. Silencing *HbM*-LO12 has been demonstrated, by studies, to reduce fungal infections and to increase the immunity to the powdery mildew fungus that infects rubber trees [74]. In *Arabidopsis thaliana*, the RIN4 protein targeted by several effectors initiates immune responses through RPM1 and RPS2. Effectors induce cell death in *Nicotiana benthamiana*, although the specific nucleotide-binding and leucine-rich repeat receptors (NLRs) involved are not known [75]. It has also been shown that the mutation of three Arabidopsis loci (MLO2, MLO12, and MLO6) diminishes powdery mildew colonization and affects interactions with other plant pathogens [76,77].

In short, powdery mildew infection is influenced by a number of factors. Bud development suppression and hyphal elongation can be attributed to premature cell wall (CW) deposition, hypersensitive reactions, and the local haustorium. Whereas traditional powdery mildew research has traditionally centered on the binary pathogen–host interaction, emerging multi-omics research emphasizes a paradigm shift: tripartite interactions among the pathogen, host, and microbiota—mediated through niche competition, ISR, and effector-microbiota crosstalk—are now recognized as key disease determinants [78,79]. This new paradigm necessitates thinking about plant defense strategies in the complexity of ecological thought.

## 3. Defense Mechanisms in Plants

### 3.1. Plants’ Defense Mechanisms Against Pathogens

**Figure 1:**
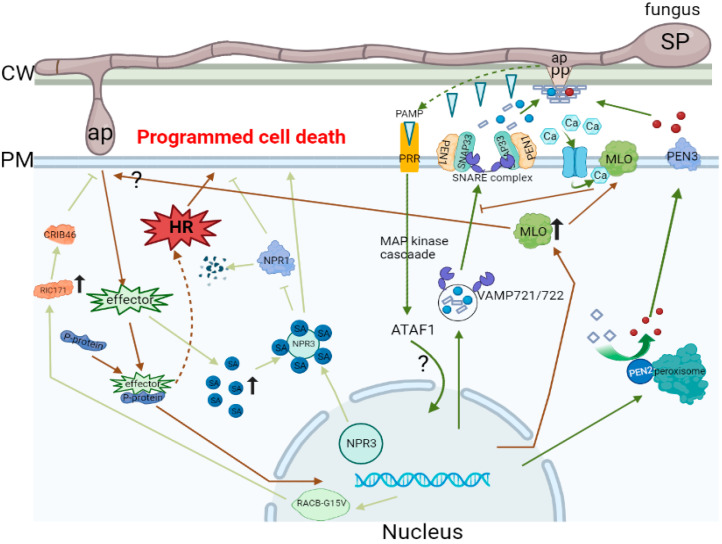
The process of plant infection by powdery mildew. i. SP attaches to the surface and forms an appressorial germ tube, which releases an enzyme, and penetration peg formation to penetrate the host layer and CW. Powdery mildew pathogenic bacteria release PAMPs and are recognized by trans-membrane pattern recognition receptors and induce MAP-kinase signaling transduction and an ATAF1-mediated invasion defense mechanism. PEN1 on the plasma membrane forms the SNARE complex with the membrane-anchoring adapter SNAP33 and the VAMP721/722 associated with the membrane compartment. The SNARE complex releases CW precursors and anti-microbial compounds (blue dots) in vesicles. The non-toxic glycoside (rhomboid) precursor is catalytic for toxic glycoside (red dot) mediated by the PEN2 enzyme and released by PEN3, thus preventing the invasion of the powdery mildew pathogenic fungus. ii. After successful invasion of the adherent cell, the effector will release the effector. The effector stimulates the cell to produce a large amount of SA, and the effector combines with P-protein to transmit the invasion signal into the nucleus. NPR3 combines with SA, which results in the inability of NPR1 to inhibit PCD, and finally the PCD occurs to resist powdery mildew. iii. The complex of P-protein and the effector may stimulate the HR and promote PCD to resist powdery mildew. iv. After the invasion signal is introduced into the nucleus, the expression of the RACB-G15V gene is upregulated, and the RIC171 in the recruitment cells gathered at the location of pathogen invasion. The CRIB46 domain of RIC171 could resist the invasion of powdery mildew. v. After the pathogen invades the cell, the expression of MLO is upregulated, and the MLO protein may open other invasion pathways for the pathogen of powdery mildew to help the pathogen invade. vi. MLO protein is located on the plasma membrane, which can regulate the level of Ca+ in cells. MLO can combine with Ca+ to drive and inhibit VAMP721/722 on the vesicle to combine with PEN1 and SNAP33 on the membrane to form the SNARE complex. The substances in the vesicle that resist the invasion of bacteria into cells cannot be released, thus helping the invasion of powdery mildew pathogens. ?: Unknown path. The arrows indicate [Red line: PIT process; Green lines: ETI process; Black lines: Pathogenic bacteria in-hibit plant defense pathways].

Plants are often subjected to an array of stress factors in the environment. Pathogenic infections may disrupt normal physiological and metabolic processes, resulting in decreased productivity and lower quality. Therefore, plants must develop defense mechanisms against an array of pests and pathogens [80,81]. In order to resist the invasion and settlement of infections, plants have acquired numerous defense layers (Figure 1). The initial layer is capable of perceiving and responding to chemicals of varied bacterial species, including non-pathogenic species. The CW is the primary defense shield against microbial disease and viruses [82]. Following the recognition of a wide range of pathogens, such as fungi and bacteria, plants anticipatively fortify their CWs through deposition as a quick response. Microbe- or pathogen-derived MAMPs/pathogen-associated molecular patterns (PAMPs) and host-derived damage-associated molecular patterns generated through attempts at infection by pathogens initiate the main line of inducible defenses [83,84]. Physical barriers, like waxes on leaf surfaces, provide an elementary level of protection. The second layer of defense attacks the virulence determinants of the pathogens directly or indirectly by modifying host targets or through antimicrobial secondary metabolites that prevent entrance by most of the plant pathogens [85]. This immune response is named pattern-triggered immunity (PTI) [86]. In addition, other effectors or elicitors are perceived by leucine-rich repeats and nucleotide-binding domains, which induce an immune response referred to as effector-triggered immunity (ETI) [87].

MAP: mitogen-activated protein; ATAF1: as a transcriptional activator, one of the first NAC proteins identified in *Arabidopsis*; PEN1, PEN2, PEN3: PENETRATION1, PENETRATION2, PENETRATION3 gene; SNARE: soluble N-ethylmaleimide-sensitive factor attachment protein receptor; SNAP33: synaptosomal-associated protein 33; VAMP721/722: vesicle-associated mem-brane protein; NPR1: non-expresser of pathogenesis-related genes 1; NPR3: non-expresser of pathogenesis-related genes 3; PCD: programmed cell death; HR: hypersensitive response; RACB-G15V: a constitutively activated RACB variant, a GTPase mutant that cannot hydrolyze GTP; RIC171: CDC42/RAC interactive binding protein of 171; CRIB46: the CRIB domain for binding of ROP-GTP, amino acid positions 23–68.

### 3.2. Mechanisms of Plant Defense Against Pathogenic Fungi

#### 3.2.1. Plants Resist Powdery Mildew Molecular Mechanism of Invasion

Conidiospores establish contact with the host plant surface, triggering the asexual life cycle of powdery mildew. Following SP germination, appressoria develop at sites where fungi try to penetrate the host CW [88]. This contact stimulates the emission of pathogen-associated molecular patterns (PAMPs), triggering PTI [89]. Successful powdery mildew isolates deploy a variety of effector molecules to bypass this initial immune reaction. Plants utilize a variety of resistance (R) genes in the achievement of ETI, which are most commonly nucleotide-binding sites and leucine-rich repeat receptors [90]. ETI has a more vigorous defense reaction than PTI [86]. Although PTI is critical for pre-invasion defense in plant–powdery mildew interactions, both PTI and ETI have significant functions in effective defense after invasion. Other plants also utilize a secondary process of defense termed programmed cell death (PCD), which often arises through an event of specific recognition between R gene products and effector proteins produced by pathogens [91]. This rapid, localized cell death effectively restricts the expansion of biotrophic pathogens, like powdery mildew [92]. MLO-mediated resistance has also been an effective strategy against powdery mildew in numerous plant species [93]. By understanding plant defense mechanisms against powdery mildew more thoroughly, we can improve disease resistance and enhance the yield potential in priority species. Apart from that, powdery mildew development regulation and bacteriostatic compound production can also be utilized in order to ease the negative effect of microorganisms on crop yields. The *Erysiphe necator* effector CSEP087 specifically interacts with grapevine arginine decarboxylase (VviADC) and suppresses polyamine biosynthesis while disrupting SA-dependent defense [14,74]. Similarly, the wheat Pm4 resistance gene generates a chimeric protein through AS that has affinity for specific effector variants and activates NLR-mediated ETI [94].

#### 3.2.2. Transcriptional Regulation for the Prevention of Powdery Mildew

Transcriptional regulation also plays a part in combating powdery mildew in plants, which is a fungal disease infecting a wide range of plant species. The potential of the plant to mount an effective defense response also seems to depend on transcriptional mechanisms. TGA functions as a transcription factor (TF) regulating defense-related gene expression. Specifically, OsTGA5 controls the rice defense-related gene expression, thereby enhancing the plant’s resistance [95]. GT also acts as a TF in the regulation of the plant growth and defense trade-off. ZmGT-3b orchestrates the metabolic pathways associated with this trade-off, compromising the gene expression involved in photosynthesis and defense in maize [96]. RNA sequencing identified the substantial activation of TF genes, including ERFs, WRKYs, MYBs, bHLHs, and NACs, that are involved in the Senna leaf defense response [97]. WRKY75 was discovered to have a positive effect on the regulation of JA-mediated defense responses against necrotrophic fungal pathogens, such as *Botrytis cinerea* and *Streptomyces* [98]. TF BcSpd1 has been associated with the downregulation of plant defense and regulation of genes participating in fungal sclerotia formation, infection buffer formation, melanin production, environmental pH modification, and fungal virulence [99]. Wheat NAC TF TaNACL-D1 participates in *Fusarium* head blight (FHB) resistance, which is economically important for FHB [100]. In addition, it has been reported that the overexpression of CaMYB39 in chickpeas enhances the accumulation of flavonoids and activates the expression of defense-related genes [101].

In brief, transcriptional regulation is important for defense against pathogen invasion.

#### 3.2.3. Powdery Mildew-Infected Plants Involved in AS Regulation

AS is the selective joining of exons from a single pre-mRNA, leading to the formation of diverse mature mRNAs [102]. This intricate process enhances genetic diversity and complexity. Moreover, AS plays a crucial role in the post-transcriptional regulation of gene expression. The regulation of AS is intricate, with the intrinsic effects of the RNA structure and variations specific to time and tissue types [103,104].

Plant pathogenic fungi employ a variety of mechanisms to manipulate plant defense mechanisms. They have different responses to chitin resistance to avoid detection [105]. Chitin deacetylase (CDA) activity plays an important role in plant defense against powdery mildew by expressing a novel gene that lacks the CDA active site, which interacts with chitin oligomers. This activity effectively blocks the activation of chitin signaling pathways [106]. To resist fungal invasion, AS is regulated in not only resistance proteins but also in the splice-associated factors of wheat plants. AS is demonstrated for the PM4 gene to produce two subtypes required for resistance activity [107]. Introna mutations in two *NtMLO* genes in powdery mildew-resistant strain Kokubu also result in abnormal splicing that enhances its resistance to powdery mildew [108]. The wheat line YK199, with its superior characteristics and resistance to powdery mildew, proved to exhibit yet another splicing form of PM4 and illustrated the regulation of the Pm4 gene. In some of the genetic stress conditions, the differential induction of splicing forms associated with the powdery mildew pathogen occurs, which increases transcriptome and protein diversity and therefore contributes to stress adaptation [17]. It has been found through research that in wheat (*Triticum aestivum*), NAC transcription factors (TaNACs) are induced by fungal pathogens based on the expression and regulation of different transcript variants [109]. The wheat aspartic protease (*TaAP*) is also alternatively spliced, with expression levels increased following powdery mildew infection. A promoter analysis has identified disease-resistant factors, like OSWRKY71 [110]. Surprisingly, pathogen effector proteins target abnormally spliced isoforms of host transcription factors specifically. They form “effector–isoform” complexes, which directly interfere with the transcriptional activation of defense genes [111]. This offers a new strategy through which pathogens subvert the host’s AS machinery to achieve immunity evasion.

## 4. Interactions of Powdery Mildew with Symbiotic Microorganism

**Figure 2:**
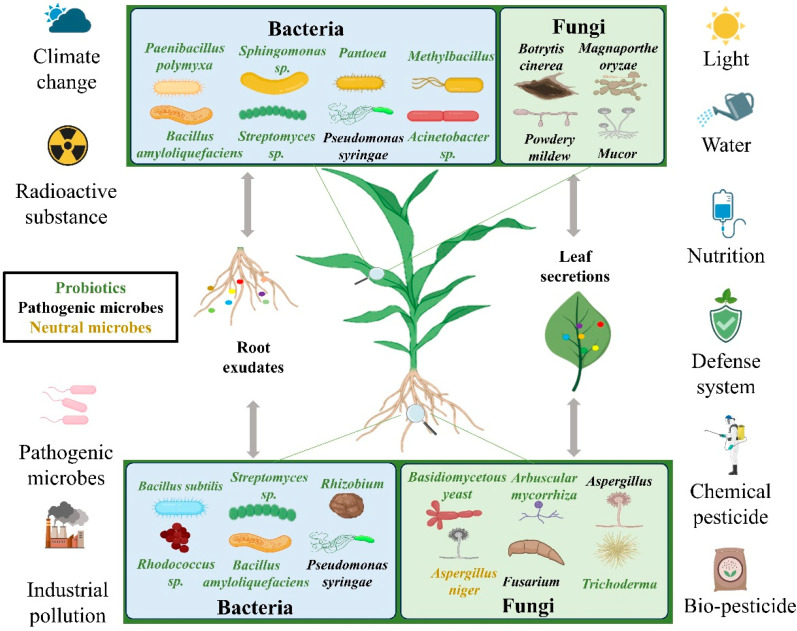
Interactions among the plant microbiome, pathogens, and environmental factors in powdery mildew dynamics. The complex interactions among plants, microbes, and the environment involve microbiome–pathogen crosstalk in which probiotics compete with powdery mildew for resources and generate antifungal compounds, environmental stresses, such as climate-driven changes in humidity and UV radiation altering leaf surface conditions that affect pathogen viability, and host–microbe synergy in which plant secretions recruit beneficial microbes and pathogens hijack host susceptibility genes (e.g., MLO) to dampen defenses. Material in this composition was sourced from BioRender.com.

Plants exist in a microorganism-rich environment. Interactions occur between the two, which can be either beneficial or harmful. Various factors influence these interactions, including the light, moisture, temperature, and human activities. These elements can modify the microbial community surrounding the plant, impacting its growth [112]. In response to threats, plants adjust the microbial structure or activate an immune response (Figure 2).

### 4.1. The Role of the Microbiome

The rhizosphere microbiome is a dynamic, selectively recruited community of microorganisms from the soil seed bank that colonizes the rhizosphere of plants [113]. The microbiome is influenced by biotic and abiotic factors and is specific to individual plant species. Surprisingly, even genotypes within a species can differ in their microbial composition. The microbial composition of the rhizosphere also varies temporally within the same plant [114,115]. This diversity can be associated with specific exudates of various genotypes or plants of a species [116]. Plants release approximately 40% of the carbon they fix through their roots into the rhizosphere, creating rhizosphere deposits. Plant root secretions, such as carbohydrates, amino acids, and organic acids, elicit a variety of reactions in the roots and are of primary significance for the organization of the rhizosphere microbiome [117,118]. Certain root exudates attract or repel specific microorganisms, thereby modifying the microbial population. Malic acid, for instance, is a tricarboxylic acid cycle metabolite released by *Arabidopsis* roots that specifically attracts beneficial *Bacillus subtilis* in the rhizosphere soil [119]. The exudates can indirectly influence the rhizosphere microbiome by modifying the physical and chemical nature of the soil. Also, they can be linked to the presence of microorganisms on and within the seeds of plants, serving as a reservoir for the microbiome [120].

In the same way, the phyllosphere microbiome is a very significant plant ecological niche [121]. Microorganisms inhabiting the phyllosphere play a crucial role in biological control. For example, *Sphingomonas* strains compete for sugars, such as glucose, fructose, and sucrose, thereby suppressing growth of the plant pathogen *Pseudomonas syringae*. A *Pantoea spp.* strain has also been reported to trigger systemic resistance, which offers defense against pests and pathogens in crops [122]. Polysaccharides of *Paenibacillus polymyxa* biofilms have also been reported to suppress *Fusarium graminearum* growth [123]. Recent research has indicated a dramatic increase in bacterial populations in pathogen-affected leaves, particularly with the rise of *Methylbacillus*, *Pantoea*, and *Acinetobacter* to varying degrees [124].

In summary, knowledge of the microbial community of the rhizosphere and phyllosphere is important for the effective control and prevention of pathogens.

### 4.2. Rhizosphere Microorganisms

The colonization of powdery mildew is controlled by a variety of microorganisms. Then, powdery mildew alters the microbial community through the secretion of some chemicals [125]. Few studies have been conducted to determine how powdery mildew affects rhizosphere microorganisms since the majority of the studies have targeted root symbiotic microorganisms. It has been observed, based on evidence, that the nodules and populations of the rhizobium in pea plants decrease under various conditions following powdery mildew infection [126]. The soils of the rhizospheres contain a variety of beneficial microorganisms that work for the well-being of the plants and stimulate fruit yields [127]. The dynamics of microbial communities in the rhizosphere are shaped by a variety of interacting factors [128]. It is important to know how these factors influence rhizosphere microbes in order to achieve successful soil ecosystem management, increase crop yields and nutrient utilization, and enhance the quality of the soil. The functional and regulatory processes governing rhizosphere microbial communities should be examined in future research, and microbial strategies for improving soil quality and sustainable agriculture should be researched.

### 4.3. Effect on the Structure of Phyllosphere Microorganisms

The process of colonization by powdery mildew is marked by the release of effector proteins by the pathogen, which interfere with the host plant immune system during invasion to enable successful colonization [129]. The aerial plant tissues, especially the leaves, are colonized by microbial communities that are regulated by a variety of biotic and abiotic factors, including the plants themselves [121]. Powdery mildew fungi infect the aboveground tissue leaves primarily, and this induces radical shifts in the composition of leaf microbial communities, resulting in significant shifts in richness and diversity [130]. Following powdery mildew infection, the foliar fungal community diversity dramatically decreases, suggesting that the invasive pathogens have a competitive advantage over most of the native leaf endophytes [5]. This competitive advantage can be due to the action of effector proteins secreted by powdery mildew pathogens, which take advantage of the indigenous endophytic fungi sharing the same ecological niche [129].

Leaf microbial community structure modification is directly linked to powdery mildew and the specific targeted host plant. Different plants exhibit host-specific associations with powdery mildew pathogens. Within this pathological situation, the severity of infection is a critical factor that decides the richness and diversity of leaf microflora [131]. Infected leaves tend to show an increase in microbial population abundance and diversity, which implies possible changes in the powdery mildew resistance of such populations. The presence of powdery mildew not only generates larger populations of bacteria but also elevates the diversity and abundance of these communities and hence affects the overall structure of bacterial communities in the leaf layer [132].

## 5. Strategies Used to Control Powdery Mildew

### 5.1. Methods of Controlling Powdery Mildew and the Need for Biological Control Agents

Today, two approaches are employed for powdery mildew control: the use of resistant plant genotypes and fungicide treatments [4]. Although the broad application of chemical pesticides is successful in the short term, it also creates significant issues with regard to environmental contamination, as well as the development of pathogen resistance [133,134,135,136]. Because of these issues, interest is turning more towards sustainable and environmentally compatible methods of powdery mildew control [137].

The use of biological control agents is a novel method for the control of this disease [138,139]. The biological control agents act as natural pathogens to the pathogen and play a vital role in reducing its number and maintaining the level of disease incidence at minimum levels. The biological control agents are made up of different types of organisms, such as useful microorganisms, such as specific bacterial and fungal isolates and parasitic insects and mites. Common biological approaches to powdery mildew control are highlighted in Table 2.

### 5.2. Biopesticides

Biopesticides, which constitute a category of agricultural antibiotics, employ living organisms or bioactive compounds derived from biological activities in the treatment of plant disease. The majority of the research on biological control today is focused on enhancing the tolerance of plants to adverse environments through chemical or physical means [145,146,147,148]. Some examples are rhizosphere bacteria for stimulating plant growth and useful strains of the fungus Trichoderma. This is fundamental for the biological control of fungal plant pathogens, thus promoting plant health, as well as guaranteeing environmental sustainability. Biopesticides are central to contemporary agriculture, providing effective disease management without the toxicity of traditional fungicides, thus guaranteeing food safety [149]. They are a major breakthrough in agricultural methods, as they can manage the disease effectively while minimizing the toxicity of traditional fungicides. Biopesticides are central to the creation of new types of pesticides, which are organized based on their structural makeup and origin, including microbial, metabolite, plant, and animal pesticides. The application of helpful microorganisms for the management of crop diseases and pests is becoming increasingly prominent. A number of biocontrol agents have been found, including *Bacillus*, *Pantoea*, and *Streptomyces* to *Trichoderma*, *Clonostachys*, *Pseudomonas*, *Burkholderia*, and a few yeasts [150]. Microbial biological control agents (MBCAs) engage in a series of biological interactions with crops for plant disease control [151]. Research shows that the application of biological control technologies in pest and disease management not only improves the efficiency but also raises economic, social, and environmental gains, in addition to addressing cost issues. Microbes are complex communities that play a crucial role in ecosystems and have impacts on plants, animals, and humans [152]. The microbial content of microbial biocontrol agents (MBCAs) is active ingredients from fungi, bacteria, and viruses that are formulated to enhance the plant environment and enhance resistance to pests and diseases. Commercial microbial agents are now sold in liquid, dry powder, and solid states. They consist of beneficial microorganisms that have different roles and utilize advanced technologies, hence enhancing high crop yields, soil fertility improvements, soil environment reconstruction, and soil micro-ecosystem reconstruction [153]. Natural ecosystems are the sources of the ingredients used in these microbial products, where decomposed matter enters back into the systems, making them sustainable, safe, and pollution-free. *Bacillus*, *Fusarium*, and *Trichoderma* have been proven to be effective in controlling powdery mildew based on research. Applications of combined microbial inoculants to crops, such as cucumbers, strawberries, and rice, are on the rise and contribute to improved crop quality and yields [103,154].

### 5.3. Environmental Impact of Fungicides vs. Biocontrol

The environmental impact of powdery mildew control is vital for the realization of sustainable agriculture. The following compares the use of chemical fungicides and emerging biocontrol tools based on their environmental impacts, addressing their long-term effects on soil health, non-target organisms, and overall ecosystem stability. To determine the environmental sustainability of powdery mildew control practices, comparing chemical fungicides with biological control agents is important. Although chemical fungicides are effective in the rapid removal of pathogens, they are also very environmentally hazardous. They can, through long-term application, detrimentally affect soil health by decreasing the microbial diversity and activity and, consequently, soil fertility and structure [155,156]. Biological control tactics are comparatively more environmentally friendly. They utilize beneficial microorganisms to suppress pathogens, thereby reducing chemical applications and enhancing soil microbial populations and ecological processes [157,158,159]. In addition, chemical fungicides are harmful to non-target organisms; for instance, broad-spectrum fungicides are toxic to beneficial insects, such as bees, thereby disrupting pollination activities in ecosystems [160,161]. Nonetheless, biological control techniques cause minimal effects on non-target species, thereby enhancing biodiversity and ecosystem stability [162,163]. Chemical fungicide overdependence results in the resistance of the pathogens, thus compromising disease management and threatening the ecological balance. However, biological control methods enhance plant resistance with the negligible development of resistance and promote long-term ecosystem stability.

In conclusion, although chemical fungicides enjoy the benefit of short-term gains by quickly reducing the population of the pathogens, biological control methods are a better prospect for long-term environmental gains and ecosystem well-being. Future practice and research must be directed towards creating and applying biological control technologies in an effort to best contain powdery mildew and facilitate sustainable agricultural progress.

## 6. Conclusions and Outlook

### 6.1. Future Directions

This review integrates three major developments in plant–microbe interaction analyses with powdery mildew as a model organism for investigating the co-evolutionary dynamics between pathogen virulence and host defense systems and between plant-biotrophs. Microbial communities that live on the phyllosphere can suppress pathogen development through the occupation of ecological niches and induction of systemic resistance and hence present a sustainable environmentally friendly alternative to conventional chemical fungicides, as represented by *Trichoderma–Bacillus* synergy [164]. The global biopesticides represent 10% of the overall pesticide market today and encompass a broad array of products from microbial pesticides to metabolites and plant- and animal-based materials [165]. Fungal effectors undermine host defense by interfering with ROS-scavenging pathways, while susceptibility genes, like MLO, collaborate with one another to govern host development. The CRISPR/Cas9 system presents significant promise in creating disease-resistance crops, particularly in the improvement of crops, such as wheat [166]. For instance, precise editing of the MLO gene in wheat using CRISPR/Cas9 has led to the creation of new strains with wide-spectrum resistance to powdery mildew [166,167]. The MLO gene has been linked to the disease susceptibility of the plant, and disabling it gives good and durable resistance [168]. In addition, this technology can be employed to downregulate or silence the expression of genes related to pest and disease susceptibility and thereby improve the natural defense system of crops or enable the development of insect-resistant genetically modified crop varieties. However, the use of CRISPR/Cas9 in disease resistance breeding is faced with various challenges, including the precision of editing, the potential for off-target effects, and long-term safety concerns. Therefore, researchers need to come up with the technology to enhance the safety and environmental sustainability of genetically edited crops and promote sustainable agriculture. In addition, transcriptional regulators and immune receptors can make context-dependent heterodimers, which offer a responsive and flexible response based on the infection phase. In Table 3, the innovations of the existing studies are compared.

### 6.2. Innovative Microbial Consortia: A Revolutionary Approach to Controlling Powdery Mildew in Agroecosystems

Its application in introducing functionally complementary microbial consortia into agroecosystems is a revolutionary approach to sustainable powdery mildew control. These consortia enhance crop resistance via two primary mechanisms: direct inhibition of the development of the pathogen through secreted antimicrobial metabolites and indirect activation of the plant systemic defense [178]. Evidence of field research points towards certain inoculants’ ability to lessen the reliance on conventional fungicides while keeping yields fixed, especially for high-value-production crops. Biopesticides are gaining traction in global agro-markets in light of stronger regulatory measures put in place on chemical fungicides, as well as the demand for environmentally friendlier ways. Projections indicate faster microbial solution adoption due to their firm edges in the aspects of environment suitability and management of residues [179]. However, scaling these technologies requires addressing critical challenges in product stability, farmer acceptance, and economic viability. Emerging methodologies in microbiome engineering and computational modeling will be instrumental in overcoming these barriers, enabling the precise manipulation of plant–microbe interactions for targeted disease control.

## Figures and Tables

**Table 1:** Studies to reveal the pathogenesis of powdery mildew and plant defense responses.

SL. No.	Study Object	Description	Refs.
1	Cytochalasins	It eliminates the polarized radial alignment of host cellular filaments at the place of CWA formation. Furthermore, it induces successful haustorium differentiation.	[29]
2	Activation of the salicylic acid (SA) pathway	The SA pathway astricts fungal growth even via a mutual effect.	[30]
3	The pmr5 and pmr6 to activate novel defenses	pmr5 and pmr6 use a similar mechanism to limit the growth of the fungus.	[31]
4	*Blumeria graminis f.* sp. Hordei (Bgh) catalase	*Bgh* catalase potentially plays a role in the removal of H_2_O_2_ produced by the host that helps *Bgh* successfully invade cells.	[32]
5	A member of the ABC transporter family is PEN3 and PDR8	PEN3/PDR8 may play a role in the export of toxins to the invasion site, and the activation of the SA pathway may be caused by the accumulation of these toxins in pen3 cells.	[33]
6	A member of the ABC transporter family is wheat LR34	A more direct role for LR34 in the resistance process may be through the export of mETEabolites that affect fungal growth.	[34]
7	The atg2-2 mutant of ATG2	Few hyphae and conidial peduncles are produced in the leaves of the ATG2-2 mutant, and a large number of foliar cells die.	[35]
8	Wheat to *Blumeria graminis f.* sp. tritici (Bgt) infection	Involvement of a defense signaling pathway mediated by the resistance gene Pm3b in triggering race-specific resistance responses to *Bgt* infection in wheat	[36]

**Table 2:** Common plant powdery mildew and control methods.

Plant Name	Prevention Method	Structure	Molecular Target and Mechanism	Refs.
Barley powdery mildew	Flutianil	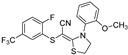	Inhibited haustorium development; affect the host cell’s haustorial formation and nutrient absorption	[140]
Hull-less pumpkin powdery mildew	Triazole fungicide	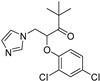	A mutation of a certain site or multiple sites ofthe CYP51 gene.	[141]
Strawberry powdery mildew	Benzothiadiazole	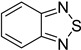	Benzothiadiazole(BTH) can enhance the accumulation of phenolics in strawberry plants, which may then be involved in the BTH-induced resistance to powdery mildew.	[142]
Rose powdery mildew	Hexaconazole	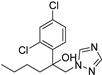	The excellent foliar efficacy of hexaconazole could be attributed to its properties of protective, eradicative, and translaminar activity with rapid speed of penetration.	[143]
Grapevine powdery mildew	Trifloxystrobin	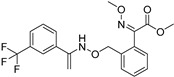	Trifloxystrobin has a partial effect on zoospore discharge and suppresses zoospore motility and the formation of germ tubes by *P. viticola*.	[144]

**Table 3:** This review compares the innovations of existing studies.

Topic	The Focus of the Existing Review	The Innovative Contributions of This Review	Key Cases
Molecular mechanisms	The immunosuppressive function of effector proteins (e.g., CSEP0064 in counteracting host RNA degradation) [169].	Dynamic AS regulation and effector protein interaction: reveals the interaction network between TF isoforms and effector proteins generated through AS to regulate ROS pathways [74,109,170].	Wheat Pm4 gene creates two resistant isoforms through AS, each recognizing a different effector protein [94,107].
Microbiome engineering	The antagonistic effect of rhizosphere microorganisms against pathogens [171,172].	The dynamics of microbial communities and the construction of synthetic microbial communities, analyzing the evolution of the microbiome during powdery mildew infection, and designing synergistic microbial communities [173,174].	The powdery mildew infection enhances the abundance of bacteria but reduces diversity [175].
Technology integration	CRISPR editing of host R genes (such as MLO knockout) [166,176].	Synchronized editing of host susceptibility genes (e.g., MLO) and the recruitment of probiotics to achieve dual regulation of “editing-microbiota”.	Gene editing and microbiome customization [177].

## Abbreviations

ROS	Reactive oxygen species
MLO	Mildew resistance Locus O
AS	Alternative splicing
SA	Salicylic acid
CWA	Cell Wall Apposition
*Bgh*	*Blumeria graminis f.* sp. *Hordei*
*Bgt*	*Blumeria graminis f.* sp. *tritici*
JA	Jasmonic acid
ET	Ethylene
NLRs	Nucleotide-binding and leucine-rich repeat receptors
PTI	Pattern-triggered immunity
ETI	Effector-triggered immunity
SP	Spore
CW	Cell wall
PM	Powdery mildew
HR	Hypersensitive response
ap	Ascospore
PAMP	Pathogen-associated molecular pattern
PCD	Programmed cell death
MAP	Mitogen-activated protein
ATAF1	As a transcriptional activator, one of the first NAC proteins identified in Arabidopsis
TF	Transcription Factor
FHB	Fusarium head blight
CDA	Chitin deacetylase activity
MAMP	Microbe-associated molecular pattern
MBCAs	Microbial biological control agents
BTH	Benzothiadiazole
